# Comparative evaluation of titanium dioxide nanoparticle reinforced resin strength

**DOI:** 10.6026/973206300212347

**Published:** 2025-08-31

**Authors:** Anuja Tripathi, Mukesh Soni, Alka Gupta, Harsh Chansoria, Varsha Mangtani, Shivani Gupta

**Affiliations:** 1 Department of Prosthodontics and Crown & Bridge, Government College of Dentistry, Indore, Madhya Pradesh, India; 2 Department of Orthodontics and Dentofacial Orthopaedics, Sri Dharmasthala Manjunatheshwara College of Dental Sciences, Dharwad, Karnataka, India

**Keywords:** Acrylic resin, impact strength, nanoparticles, tensile strength, titanium dioxide (TiO_2_)

## Abstract

The impact of varying concentrations (0.5%, 1% and 5%) of titanium dioxide (TiO_2_) nanoparticles on the tensile and impact strength of heat-cured, reinforced, colour-modified acrylic resin. The data showed that TiO_2_ nanoparticles significantly enhanced the mechanical properties of the material, with 1 wt% TiO_2_ providing the highest tensile strength and 5 wt% TiO_2_ showing the highest impact strength. Thus, we show that TiO_2_ nanoparticles are promising for improving the durability of acrylic resins in denture applications. However, nanoparticle agglomeration at higher concentrations may reduce mechanical performance, warranting further research on dispersion methods.

## Background:

Apart from suitable mechanical and physical characteristics, a good denture base material should exhibit biocompatibility and aesthetic appeal [1]. Fundamental mechanical properties of the material, if it is to resist and sustain various forces applied during normal operation, are tensile strength and impact strength [2]. The largest stress a material can withstand before breaking is defined by tensile strength, which is the internal force opposing material elongation along the direction of the applied tension [3]. Conversely, impact strength is the force required to break a material. The ideal denture base must be able to resist both dynamic and static stresses generated by chewing and other mouth movements [4]. Mostly used in denture base construction, polymethyl methacrylate (PMMA) is chosen for its economy, simplicity of manufacture, biocompatibility and great cosmetic qualities [5]. It has several restrictions, but particularly in terms of its mechanical properties. Its reduced hardness and surface wear affect its aesthetic durability and can cause microbial attachment, therefore aggravating conditions such as denture stomatitis [6]. Moreover, PMMA is more likely to exhibit impact fractures, bending deformation and fatigue due to dynamic pressures. These defects unequivocally illustrate how much the mechanical resilience of the material influences the lifetime and the degree of superior performance that dental applications depend on [7]. These limitations have driven several projects aiming at raising PMMA performance criteria. Combining molecules increases the crosslink density in the polymer matrix. Studies on additions such as polyethene glycol dimethacrylate seek to maximize the molecular architecture and thereby improve the mechanical properties of the material including fibre or particle reinforcements into the acrylic resin presents still another fascinating approach. Typically, when laying the denture foundation, these reinforcements prevent stress transfer agents from spreading cracks [8].

Although traditional techniques of strengthening have focused on adding micrometre-sized fillers to boost strength and resistance to deterioration, these fillers can also increase material brittleness and reduce optical translucency [9]. As such, the development of nanocomposites generates one original solution. Where at least one filler dimension is less than 100 nm, nanocomposites where the mechanical properties of PMMA can be greatly enhanced without compromising appearance, can be rather successful [5]. Using titanium dioxide (TiO_2_) nanoparticles has been shown to improve the durability, fracture toughness and fatigue resistance of dental prosthesis [10]. Because of its remarkable surface hardness, biocompatibility, chemical inertness and antibacterial action, especially TiO_2_ nanoparticles draw interest in dental materials [11]. TiO_2_ nanoparticles enhance PMMA's mechanical properties, which increases its wear and damage resistance according to studies on them [12]. Through increased opacity and thereby reduced bacterial colonization, TiO_2_ nanoparticles may improve the visual appeal of the material and thereby prevent denture-related disorders [13]. Though these advantages abound, adding TiO_2_ nanoparticles to PMMA, there are several challenges. High TiO_2_ values could affect the colour of the resin, thereby affecting its appearance and leaving a negative impression [14]. Maintaining equilibrium between desired aesthetic values and improved mechanical performance thus requires regulating the concentration and dispersion of TiO_2_ nanoparticles [15]. Since TiO_2_-based Nano composites for dental applications are still in their early years, more research is needed to fully understand how TiO_2_ nanoparticles influence the mechanical and aesthetic qualities of PMMA [16]. This paper evaluates the tensile and impact strength of heat-cured, reinforced, colour-modified acrylic resin at several TiO_2_ nanoparticle concentrations (0.5%, 1% and 5%). Therefore, it is of interest to demonstrate the expected capacity of TiO_2_ nanoparticles in enhancing the mechanical properties of PMMA, thereby improving its long-term performance in denture applications.

## Methodology:

The study is a prospective, experimental, comparative and quantitative and *in-vitro* investigation conducted at the Department of Prosthodontics and Crown & Bridge, Government College of Dentistry, Indore, with tensile and impact strength testing carried out at KAILTECH Test & Research Centre Pvt. Ltd., Indore. A total of 80 rectangular bar-shaped specimens (n=80) were fabricated with final dimensions of 50 mm in length, 10 mm in width and 3 mm in thickness. These specimens were created using heat-cured reinforced color-modified acrylic resin with varying percentages (0.5%, 1% and 5%) of TiO_2_ nanoparticles. The control group (Group 1) contained reinforced color-modified acrylic resin without TiO_2_ nanoparticles. For the other groups, appropriate amounts of titanium dioxide nanoparticles (0.5 wt%, 1 wt% and 5 wt%) were mixed with the resin polymer and monomer in a 2:1 weight ratio during the dough stage, followed by packing the mixture into molds and polymerizing it. The tensile and impact strength of these specimens were tested using a Universal Testing Machine and Charpy's Impact Tester, respectively. The materials used in the study included heat-cure acrylic denture base resin, TiO_2_ nanoparticles, color pigment, dental stone and modeling wax, while equipment included a Universal Testing Machine, Charpy's Impact Tester, micromotor handpiece, acrylization unit, bench press, dewaxing unit and other necessary tools. All specimens were processed, finished and polished according to standard procedures before testing, with each group consisting of 20 samples-10 used for tensile strength testing and 10 for impact strength testing. The samples were evaluated for dimensional accuracy using a digital vernier caliper and polished to eliminate any surface imperfections. Testing was carried out to determine the tensile and impact strength of the specimens and results were recorded and compared across the different groups. The study aimed to assess the effect of varying TiO_2_ concentrations on the mechanical properties of color-modified acrylic resin to improve prosthetic longevity and functionality in dental applications. Data were entered into an Excel sheet and analyzed using SPSS (Statistical Package for Social Sciences) version 25.0. The Kolmogorov-Smirnov test was used to check the probability distribution of the data, which was found to be normally distributed. The data were presented as mean ± standard deviation. Inter-group comparisons were done using One-way ANOVA, followed by post hoc analysis using Tukey's test. A p-value of <0.05 was considered statistically significant.

## Formulae used:

Mean (x-): The mean is calculated by dividing the sum of the individual data points by the total number of observations (n).

Standard Deviation (SD): The standard deviation is used to describe the variability of a data set, measuring how spread out the data points is around the mean.

## One-way Analysis of Variance (ANOVA):

The F-test statistic for one-way ANOVA is calculated by comparing the between-group variability and within-group variability. Post-hoc tests were performed using Tukey's Honest Significant Difference (HSD) method, which is used after an ANOVA to compare all possible pairs of means. Where M represents the treatment or group mean, S_W is the standard error and n is the number of observations per group. The null hypothesis for this study is that the addition of varying percentages of TiO_2_ nanoparticles has no significant effect on the tensile and impact strength of colour-modified acrylic resin compared to conventional acrylic resin.

The alternate hypothesis for this study is that the addition of TiO_2_ nanoparticles significantly affects the tensile and impact strength of colour-modified acrylic resin compared to conventional acrylic resin.

## Results:

The study demonstrated that the addition of TiO_2_ nanoparticles significantly enhanced both tensile and impact strength in reinforced color-modified acrylic resin Table 1 (see PDF) Tensile and Impact Strength Values shows the values for tensile and impact strength in all the groups. Group 3 (1 wt% TiO_2_) exhibited the highest tensile strength, followed by Group 4 (5 wt% TiO_2_), Group 2 (0.5 wt% TiO_2_) and Group 1 (control group with no TiO_2_). Group 3 had a significantly higher tensile strength compared to all other groups (p-value < 0.05) Table 2 (see PDF) Inter-group Comparison of Tensile Strength highlights the statistical comparison between the groups, confirming that the differences between Group 3 and the others were significant. Post hoc analysis of Tensile strength has been shown in Table 3 (see PDF). For impact strength, Table 4 (see PDF) Inter-group Comparison of Impact Strength shows that Group 4 (5 wt% TiO_2_) achieved the highest impact strength, followed by Group 3, Group 2 and Group 1 Table 5 (see PDF). Post-hoc Analysis (Impact Strength) provides a detailed analysis of the differences in impact strength between the groups. Group 4 had significantly higher impact strength than the other groups (p-value < 0.05). The Graphs 1 and 2: Tensile and Impact Strength in Groups 1, 2, 3 and 4 further visualize these results, with Figure 1 (see PDF) illustrating the variation in tensile strength and Figure 2 (see PDF) showcasing the differences in impact strength among the groups. These results highlight the positive impact of TiO_2_ nanoparticles on enhancing the mechanical properties of the material, particularly in terms of tensile and impact strength. The tensile strength was highest in Group 3, followed by Group 4, followed by Group 2, followed by Group 1. Group 3 > Group 4 > Group 2 > Group 1 The tensile strength in Group 3 was significantly greater than that in Group 4, Group 2 and Group 1 (p-value <.05). The impact strength was highest in Group 4, followed by Group 3, followed by Group 2, followed by Group 1. Group 4 > Group 3 > Group 2 > Group 1 The impact strength in Group 4 was significantly greater than that in Group 3, Group 2 and Group 1 (p-value <.05).

## Discussion:

Polymethyl methacrylate (PMMA) is primarily utilized in denture base fabrication due to its cost-effectiveness, ease of processing, biocompatibility and favorable aesthetic characteristics [16]. However, its mechanical limitations have prompted extensive research aimed at enhancing its performance. One strategy involves the incorporation of crosslinking agents, such as polyethylene glycol dimethacrylate, to increase the crosslink density within the polymer matrix and optimize the molecular structure, thereby improving mechanical strength. Another promising approach involves reinforcing PMMA with particles or fibers. These reinforcements serve to intercept stress propagation within the material, thereby reducing the risk of crack formation and propagation in denture bases [17]. Traditional reinforcement techniques have often relied on the inclusion of micrometer-sized fillers to improve resistance to mechanical degradation. However, such fillers may inadvertently increase material brittleness and diminish optical translucency, limiting their clinical appeal. In contrast, nanocomposite technology offers a novel and promising solution. When at least one dimension of the filler is below 100 nm, nanocomposites can significantly enhance the mechanical properties of PMMA without compromising its aesthetic qualities [18]. Among these, TiO_2_ nanoparticles have demonstrated the ability to enhance durability, fracture toughness and fatigue resistance in dental prostheses [19]. TiO_2_ nanoparticles are particularly attractive in dental materials due to their superior surface hardness, chemical inertness and biocompatibility and antimicrobial properties. Their incorporation into PMMA has been shown to improve wear resistance and structural integrity while simultaneously reducing microbial colonization due to increased surface opacity. These enhancements contribute to both functional performance and the prevention of prosthesis-related complications, such as denture stomatitis [20]. Despite these benefits, certain challenges persist. High concentrations of TiO_2_ nanoparticles may negatively impact the optical properties of PMMA, particularly its color, leading to undesirable aesthetic outcomes. Therefore, achieving an optimal balance between improved mechanical properties and acceptable visual appearance necessitates careful control over the concentration and homogeneous dispersion of TiO_2_ nanoparticles within the resin matrix [21].

## Conclusion:

The incorporation of TiO_2_ nanoparticles significantly enhanced the tensile and impact strength of reinforced color-modified acrylic resin, with 1 wt% TiO_2_ showing the highest tensile strength and 5 wt% TiO_2_ showing the highest impact strength. Thus, we show the potential of TiO_2_ as a reinforcing agent for PMMA. Further research and advancements in 3D printing could optimize TiO_2_ nanoparticle incorporation for patient-specific prosthetic designs.
